# Emerging hotspots of groundwater conflicts during droughts in Germany

**DOI:** 10.1016/j.isci.2026.116338

**Published:** 2026-06-11

**Authors:** Jan Sodoge, Christian Kuhlicke, Giuliano Di Baldassarre, Jan H. Fleckenstein, Pia Ebeling, Mariana Madruga de Brito

**Affiliations:** 1 Department of Urban and Environmental Sociology, Helmholtz Centre for Environmental Research (UFZ), Leipzig, Germany; 2 Technopolis Group, Frankfurt, Germany; 3 Institute of Environmental Science and Geography, University of Potsdam, Potsdam, Germany; 4 Department of Earth Sciences, Uppsala University, Uppsala, Sweden; 5 Department of Hydrogeology, Helmholtz-Centre for Environmental Research (UFZ), Leipzig, Germany; 6 Hydrologic Modelling Unit, Bayreuth Center of Ecology and Environmental Research (BayCEER), University of Bayreuth, Bayreuth, Germany; 7 Centre of Natural Hazards and Disaster Science (CNDS), Uppsala, Sweden

**Keywords:** earth sciences, environmental science, environmental monitoring

## Abstract

Groundwater conflicts are increasing worldwide, particularly during droughts. While conflict dynamics have been widely studied in historically water-scarce regions, emerging hotspots in Europe remain understudied. We introduce a text-mining approach to monitor conflicts over time and apply it to Germany, where droughts have exacerbated tensions among water users. Using a corpus of over 12,000 news articles published between 2000 and 2022, we map the spatiotemporal distribution of reported conflicts and identify their drivers using a metric of reported conflict intensity combining frequency and prominence of conflict reporting. Groundwater conflicts are geographically widespread, with recurring hotspots and new conflict areas emerging during the 2018–2022 multi-year drought. While water pollution and environmental protection have diminished in importance, scarcity, agriculture, and drought have become key drivers. Reported conflicts show only moderate spatial correlations with groundwater withdrawal, recharge, and pollution, suggesting media dynamics and social context shape which conflicts become publicly visible.

## Highlights

• Groundwater conflicts in Germany increased, forming four recurring regional hotspots
• The 2018–2022 drought triggered new conflict hotspots in northeastern Germany
• Scarcity and drought replaced pollution as dominant reported conflict drivers
• Text mining enables scalable monitoring of non-violent groundwater conflicts

## Graphical abstract

**Figure:**
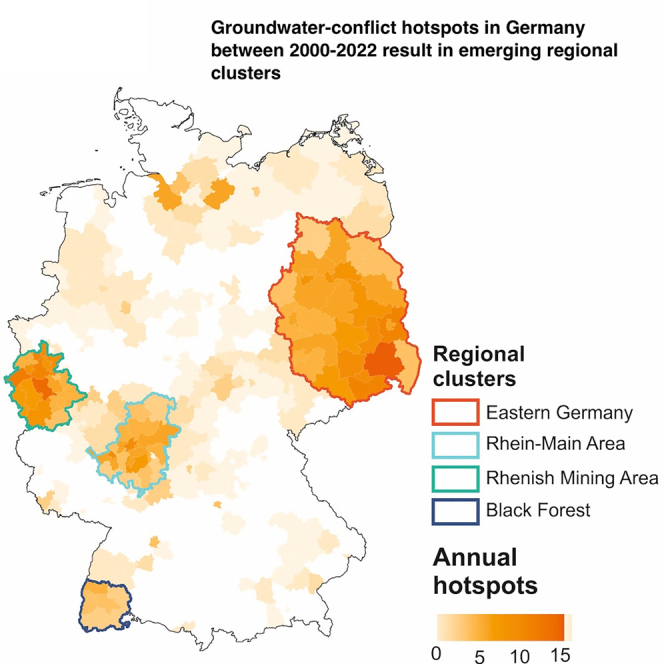

## Introduction

Conflicts involving water are increasing on a global scale, ranging from regional disagreements over water allocation to international geopolitical conflicts.^1^^,^^2^^,^^3^ Since 2020, the Water Conflict Chronology reported 785 cases of conflicts worldwide, accounting for 40% of the database coverage.^4^ While surface water resources are increasingly pressured by climate change and anthropogenic influences such as competing demands and pollution, groundwater extraction is also giving rise to new conflicts over groundwater resources.^5^^,^^6^ These groundwater-related conflicts pose unique challenges due to the hidden nature of groundwater systems, their uncertain state, and the complexity of the interconnected hydrological systems.^7^^,^^8^

Although many disputes over water resources distribution are non-violent,^9^^,^^10^^,^^11^ researchers have mostly focused on violent cases, with few studies addressing non-violent forms of conflict.^12^^,^^13^ This focus is mirrored in key databases, such as the Water Conflict Chronology^14^ and the Transboundary Freshwater Disputes Database,^15^ both of which emphasize violence as a defining feature of conflicts. However, groundwater-related conflicts in Central Europe illustrate a different reality. Here, they are often characterized by competing stakeholder interests and views, rather than physical violence.^16^^,^^17^^,^^18^^,^^19^ This underscores the need for research focused on the dynamics of non-violent groundwater conflicts.

A further research gap is that research has predominantly focused on regions with drier climates and long-standing water disputes often related to water scarcity.^2^^,^^10^^,^^14^^,^^20^^,^^21^^,^^22^ For example, a global review documented 86 water-related conflicts in the Middle East between 1951 and 2019, compared to just 18 in Europe.^2^ As a result, newly emerging groundwater conflicts in temperate regions, such as Central Europe e.g., Brauner et al.^16^ and Kosow et al.,^19^ have received limited attention.^23^ Research indicates that droughts or arid conditions alone do not drive these disputes. Instead, they arise from a complex interplay of drivers, with drought being a key contributing factor but not the sole one. In fact, drought has been found to exacerbate conflicts mainly when combined with pre-existing socio-economic stressors or when it increases the exposure of water-dependent users to resource scarcity. For instance, when declining groundwater levels coincide with high irrigation demand or constrained public water supply.^9^^,^^10^^,^^24^

This interplay of climatic and socio-economic pressures is particularly relevant in Central Europe, for instance in Germany, where increasingly frequent droughts paired with high water demands have heightened water stress^25^^,^^26^ and triggered new conflicts.^27^^,^^28^^,^^29^ Drought periods since 2018 imposed unprecedented pressure on the German groundwater system, which supplies more than 70% of the country’s drinking water.^30^^,^^31^ This pressure is expected to worsen in the future as climate projections point to potential further declines in groundwater levels.^26^^,^^32^ At the same time, agricultural irrigation demand is expected to rise,^19^^,^^33^^,^^34^ increasing the exposure of irrigated agriculture and public drinking water supply systems to groundwater scarcity. For instance, when more frequent irrigation restrictions increase the risk of related conflicts. These tensions are already reflected in the growing number of legal disputes over groundwater across Germany, with cases in Bavaria (federal state in southern Germany) nearly doubling between 2018 and 2022.^35^ Together, these trends underscore the urgency of addressing groundwater-related conflicts in emerging European hotspots.

In this paper, we address these gaps by introducing a scalable method to systematically identify non-violent conflicts across large text corpora, overcoming the limitations of traditional small-N case-study approaches and capturing disputes often missed by conventional conflict analyses. Using a text-mining approach, we examine the extent to which groundwater conflicts have been reported in German news from 2000 to 2022. More specifically, we investigate (1) the spatial and temporal dynamics of groundwater-related conflicts and their underlying causes, (2) the influence of the 2018–2022 multi-year drought on conflict patterns, and (3) the potential link between reported conflicts and groundwater withdrawal and recharge rates (Table 1). By shedding light on emerging groundwater conflicts in Germany, we provide an approach for quantifying water-related disputes beyond the traditionally studied arid regions.

**Table 1:** Overview of groundwater-related variables: original temporal-spatial resolution and sources

Variable [unit]	temporal resolution	spatial resolution	source
Public and non-public groundwater withdrawal [1,000 cubic meters]	3-year intervals (1998–2022)	NUTS-2	Statistisches Bundesamt^70^
Groundwater recharge [mm/yr]	long-term average over the period 1961-1990	1 × 1 km^2^	Bundesanstalt für Geowissenschaften und Rohstoffe^71^
Nitrate concentration [mg/L]	single measurement (2016–2018)	well-based	Umweltbundesamt^72^

## Results

### CMD as a tool for detecting conflicts and underlying drivers

**Figure 1:**
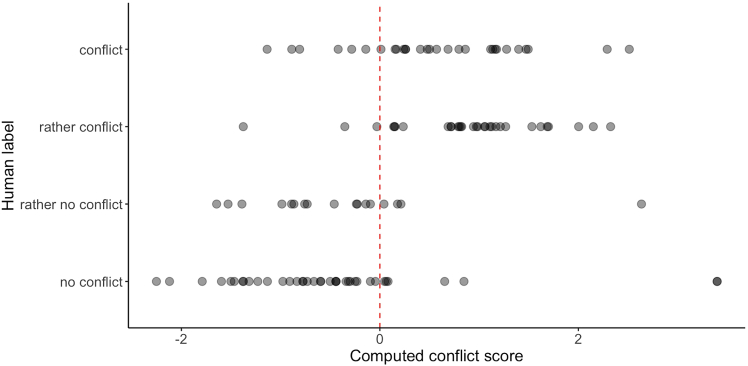
Evaluation of conflict classification using CMD Evaluation for the classification of groundwater-related conflicts using concept movers distance (CMD). The CMD for “groundwater conflict” is compared to 144 annotated texts from randomly sampled news articles.

Using our natural language processing (NLP)-based text-mining approach, we identified 4,823 unique news articles reporting on groundwater-related conflicts in Germany from 2000 to 2022 across a large corpus of >12,000 articles (Figure S1). Among these, 3,205 (66%) provided information on the conflict location. The classification accuracy of the concept movers distance (CMD) approach reached 0.8 for separating articles labeled “no conflict” and “conflict” on an evaluation sample of 144 articles (Figure 1). When including articles labeled “rather no conflict” and “rather conflict”, the accuracy increased to 0.82. The ambiguities in determining whether articles report on conflicts are reflected in the inter-annotator agreement (Krippendorff’s Alpha) of 0.90. Importantly, 78% of disagreements occurred between adjacent categories, either no conflict and rather no conflict or conflict and rather conflict. This ambiguity highlights the value of the continuous CMD measure, which captures the nuanced spectrum of conflict intensity more effectively than discrete labels.

The proposed zero-shot topic modeling approach also proved effective in identifying the reported conflict drivers (Table 2). Classification accuracy was high across all drivers, ranging from 0.93 for “drinking water supply” to 0.85 for “water quality”. The models also achieved high precision (median = 0.96), indicating that most of the identified drivers were indeed mentioned in the article’s text. Recall was slightly lower (median = 0.89), suggesting that the model misses some relevant drivers mentioned in the text. However, this limitation is mitigated by the fact that conflict drivers are often reported repeatedly within the same article. In fact, for 42% of articles, we find at least one driver reported more than once.

**Table 2:** Assessed drivers influencing groundwater-related conflicts and their respective classification performance metrics

Category	Description	Accuracy	Precision	Recall
Drought	Prolonged dry periods can lead to groundwater depletion, intensifying conflicts over scarce water resources	0.92	0.98	0.91
Agriculture	Over-extraction of groundwater for irrigation, particularly in water-scarce regions	0.87	0.90	0.93
Mining and other industry activities	Mining operations can disrupt groundwater availability and quality. Industrial activities often require large quantities of groundwater	0.81	0.81	0.92
Drinking water supply	Supplying drinking water to the population demands groundwater resources and competes with other users	0.83	0.93	0.85
Environmental protection of groundwater resources	Environmental protection and environmental regulations of groundwater bodies or ecosystems can result in conflicts over the involved groundwater resources	0.83	0.85	0.90
Water quality	Pollution from various sources (e.g., industry, agriculture) degrades groundwater quality, resulting in conflicts over access to safe water	0.87	0.93	0.91
Groundwater scarcity	Persistent low groundwater availability	0.91	0.95	0.94

Each model was evaluated on 100 observations.

### Groundwater conflict hotspots intensify and spread across Germany

**Figure 2:**
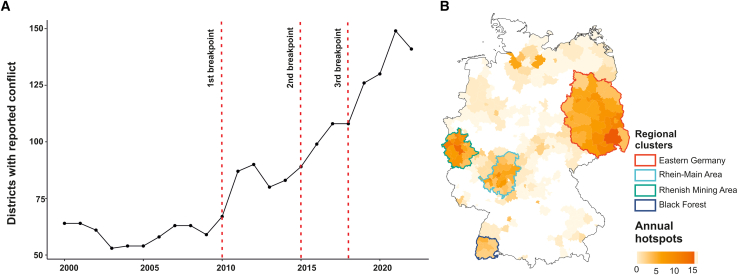
Spatial and temporal spread of groundwater conflict hotspots across Germany (A) Number of districts in Germany with at least one reported groundwater conflict. Dashed lines indicate breakpoints identified. (B) Annual number of hotspots per district, with recurrent regional hotspot clusters highlighted.

Overall, the spatial extent of groundwater-related conflicts grew rapidly across Germany between 2000 and 2022 (Figure 2A). The number of districts reporting at least one conflict more than doubled, from 64 to 141 (out of 401), indicating a broad diffusion beyond early hotspots, particularly after 2010. This coincided with a sharp decrease in the number of districts classified as coldspots (i.e., those with low conflict levels) from 221 in 2000 to just 15 in 2022. Time-series analysis revealed three breakpoints signaling shifts in conflict reporting: 2010, 2015, and 2018. These years mark moments when the trajectory of conflict reporting changed more drastically.

Results of the Getis-Ord Gi∗ hotspot analysis revealed an uneven distribution of reported conflicts, with persistent regional hotspots where conflict reporting is concentrated (Figure 2B). This is reflected in an unequal distribution of conflict-related articles: while most districts report few conflicts, a small subset of the districts accounts for a disproportionate share, with the 10 most affected districts accounting for 20% of all reported conflicts (see Figure 2B). For example, district Bautzen in Eastern Germany was a hotspot in 15 of the 23 analyzed years, indicating sustained localized pressures. At the same time, large parts of the country have remained coldspots for most of the study period. In fact, 64% of the districts occur in a conflict hotspot at most once, indicating that conflict hotspots are singularities rather than recurrent phenomena in most districts.

The districts with recurring groundwater conflict hotspots cluster into four regions: (1) eastern Germany, (2) the Rhine River Valley near the German-Dutch border, (3) the greater Main area around Frankfurt, and (4) the south-western Germany along the Upper Rhine area (Figure 2B). Among these, the largest cluster is in Eastern Germany, spanning 29 districts. Across the analyzed period, only in 2003 no conflict hotspots occurred in this cluster, while on average, a median of 9 districts per year were involved in this area. Other clusters show more variable patterns. The Main area hotspot cluster, for example, shows increased activity during both the early years (2000–2004) and the end of the study period (2018–2022), while hotspot occurrence declined between 2005 and 2014. The southwestern Germany cluster reached its peak spatial extent between 2005 and 2010 and then steadily declined toward the end of the study period. None of the clusters maintained a stable size throughout the analyzed years; instead, all showed considerable yearly variability in the number of affected districts within each cluster, reflecting fluctuations in media attention to conflicts over time. We use these regional clusters in the subsequent sections to describe and compare regional conflict patterns in Germany over time.

### The shifting drivers of groundwater conflicts in Germany

**Figure 3:**
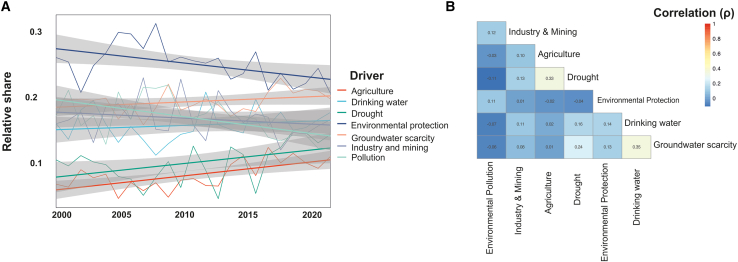
Temporal development of reported conflict drivers (A) Temporal development of the relative share of drivers over time. (B) A linear trend is displayed for each driver next to the observations.

The prominence of the seven groundwater conflict drivers in news reporting changed over time (Figure 3A). Agriculture and drought became increasingly prominent. Conversely, environmental protection and pollution declined steadily. Despite these shifts, year-to-year variability remains high, indicating that the drivers of reported conflicts fluctuate substantially over time rather than following smooth trajectories. In fact, the remaining drivers (drinking water, groundwater scarcity, industry, and mining) showed no significant long-term trends.

**Figure 4:**
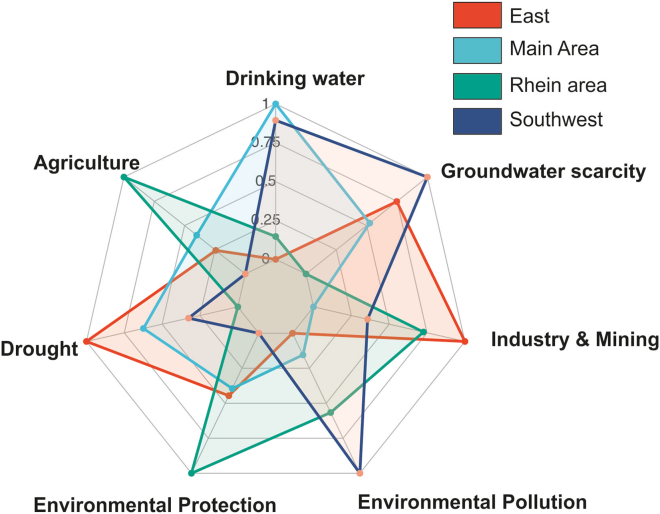
Regional variation in conflict drivers across hotspot clusters Distribution of drivers’ relative contributions across each regional cluster. The displayed distribution is based on the annual data for each respective region. An observation is included for a regional hotspot cluster only if a hotspot was identified within that region during the corresponding year.

Conflict drivers varied largely across the four regional clusters. South-western Germany had the highest levels of articles mentioning “pollution” as a conflict driver, while “drought” and “agriculture” were less mentioned (Figure 4). In contrast, drought is the leading driver of conflicts in eastern Germany and the Main area. The river Rhine cluster, however, followed a different pattern: neither drought nor pollution was particularly dominant. Instead, this cluster showed the highest relative share from agriculture and “environmental protection” drivers. These heterogeneous patterns are not static. They evolve dynamically over time. For example, in the south-western cluster, the pollution driver declined steadily over the years, and a similar decrease was observed for environmental protection in the Rhine cluster. In other clusters, however, both drivers remained consistently low and showed little change. Compared with non-hotspot districts, cluster regions show a significantly higher reporting of drought (*p* = 0.008), industry and mining (*p* = 0.01), and pollution (*p* = 0.01), whereas agriculture appeared significantly more often as a driver (*p* = 0.05) in non-cluster districts.

Moran’s I values indicated that drought and “drinking water” were the spatially most concentrated drivers (0.22–0.24), suggesting they tend to cluster in specific locations rather than being evenly distributed across the country (Figure S3). In contrast, drivers such as “groundwater scarcity” and environmental protection were evenly distributed across Germany, with no clear spatial concentration. Beyond single drivers, certain drivers tend to compound one another: drinking water issues frequently were reported together with groundwater scarcity (*ρ* = 0.35), suggesting shared underlying pressures on local water resources. Similarly, agriculture and drought often overlapped (*ρ* = 0.33), as did groundwater scarcity and drought (*ρ* = 0.24), potentially indicating mutually reinforcing stressors. This compounding effect was particularly pronounced in the Eastern Germany cluster, where districts reporting drought were also likely to report agriculture as a key driver (*ρ* = 0.50). These co-occurrence patterns similarly reflect their conceptual proximity. For instance, drought and groundwater scarcity describe partially overlapping phenomena, while agriculture and drought frequently compound one another as reported pressures on groundwater resources.

### Drought periods are linked to new hotspots of groundwater conflicts

**Figure 5:**
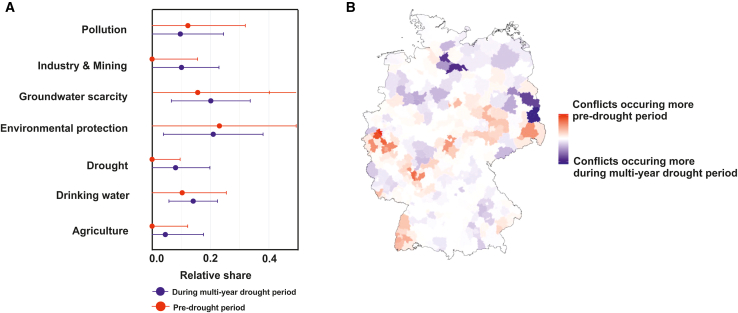
Changes in conflict drivers and spatial distribution during the 2018–2022 multi-year drought (A) Changes in the relative prevalence of the different drivers before (2000–2017) and during the multi-year drought (2018–2022). (B) Changes in the spatial distribution of reported conflicts before and during the multi-year drought.

We examined how and where droughts were associated with increases in reported groundwater conflicts. Our analysis revealed that the 2018–2022 multi-year drought coincided with higher conflict intensity in some regions (Figure 5B). The greatest increases occurred in the greater Hamburg area and northeastern Germany. Hamburg recorded the highest conflict levels nationwide compared to the pre-drought years, while the surrounding district of Lüneburg recorded the third-largest increase. A second emerging hotspot during the drought period appeared in northeastern Germany near the German-Polish border, where two districts exhibited the strongest increases in reported conflicts between the pre-drought and drought periods. At the same time, conflicts in south-western Germany and the Rhine region became less prevalent: during the multi-year drought, south-western Germany had 0 hotspot districts, and the Rhine region had 1.

Alongside new spatial hotspots, we detected significant changes in the reported conflict drivers between the pre- and post-multi-year drought (Figure 5A). During 2018–2022, there were significant increases in the reporting of agriculture (Wilcoxon test, *p* = 0.007), drought (*p* < 0.001), groundwater scarcity (*p* = 0.01), and drinking water-related conflicts (*p* = 0.007) compared with preceding years. Regionally, these changes manifested in distinct ways. In the greater Hamburg area, groundwater scarcity became a significantly more prominent driver than in districts elsewhere in Germany (*p* = 0.002). In Eastern Germany, industry and mining were reported more frequently as drivers, although these increases were not statistically significant.

### Groundwater withdrawal rates correlate with conflict intensity

Reported groundwater-related conflicts were moderately associated with physical groundwater indicators (Table 3), with the strongest links observed for non-public groundwater withdrawal. The volume of non-public groundwater extraction showed moderate positive correlations (*ρ* = 0.44), indicating that districts with higher levels of non-public groundwater use are more likely to report groundwater-related conflicts. Specifically, districts in the highest quantile (75^th^) of reported conflicts display higher average non-public withdrawal volumes (mean = 123.4 × 10^3^ m^3^) than the remainder of districts (mean = 33.3 × 10^3^ m^3^). Next to the total volume, the proportion of non-public withdrawal among all extractions is also moderately correlated with conflict occurrence in the non-public sector (*ρ* = 0.46). Public groundwater withdrawals were less strongly associated with conflicts (*ρ* = 0.29). However, this link increased during the multi-year drought (*ρ* = 0.40), indicating that during this period, the reporting on conflicts was more closely linked to public groundwater withdrawal than before. Combined total withdrawals (i.e., public and non-public extractions) also showed stronger correlations during drought (*ρ* increased from 0.45 to 0.52), reflecting the cumulative pressure on aquifer systems.

**Table 3:** Correlation coefficients (Spearman’s Rho *ρ*) between reported conflicts per NUTS-2 district and selected variables reflecting groundwater quantity and quality

Indicator	Reported conflicts	Reported conflicts multi-year drought
Public withdrawal volume	0.29	0.40
Public withdrawal share	−0.08	0.18
Non-public withdrawal volume	0.44	0.42
Non-public withdrawal share	0.46	0.19
Combined withdrawal volume	0.46	0.52
Groundwater recharge	−0.02	−0.00
Nitrate concentration	0.45	0.52

Values with *ρ* ≥ 0.3 indicate moderate and *ρ* ≥ 0.5 strong correlations.

Neither groundwater recharge nor nitrate concentration displayed a consistent relationship with reported conflicts. For groundwater recharge, only in some districts, such as Brandenburg (eastern Germany) and Darmstadt (in the Main area), displayed compounding low recharge rates with high conflict volumes. Similarly, no significant correlation was found between reported conflicts and nitrate concentrations in groundwater (*ρ* = −0.01). Nevertheless, nitrate levels show a moderate correlation with the proportion of pollution as a reported driver (*ρ* = 0.30). In particular, in Lower-Saxony, we found an increased reporting of pollution as a driver, which matches a high number of districts in this region with above threshold NO_3_ pollution. One example is Braunschweig (northern Germany), where pollution is frequently reported as a driver and nitrate concentrations are high. Yet, conflict levels in this district remain low, indicating that groundwater quality degradation alone does not necessarily translate into heavily reported conflicts.

## Discussion

Groundwater conflicts, long associated with drier regions, are now emerging across Europe. Yet, systematic research on their occurrence and drivers within the European context remains limited.^19^ Recent studies identify Germany as an emerging hotspot of groundwater tensions e.g., Stein and Tröltzsch^27^ and Joeres et al.,^35^ where consecutive droughts have stressed water resources^26^ and led to a rise in legal disputes over groundwater use.^35^ Therefore, quantifying and understanding how and where these conflicts emerge is critical for anticipating future crises, particularly in regions traditionally assumed to be water-secure. Therefore, we provide new evidence on the evolution of groundwater conflicts in Germany since 2000 by combining large-scale text mining with systematic analysis of news media coverage. Our approach focuses specifically on conflicts that have gained public visibility through media reporting, thereby capturing disputes that transcend private disagreements to become matters of public concern and debate.^36^ In doing so, we contribute to this ongoing debate by (1) demonstrating how non-violent groundwater conflicts can be quantified with text-mining, (2) identifying regional conflict hotspot clusters and their drivers, (3) capturing shifts in conflict-reporting driven by the 2018–2022 multi-year drought as well as groundwater resource conditions.

Our analysis shows a clear rise in the prevalence of reported groundwater-related conflicts in Germany since 2000, affecting an increasing number of districts. Our findings align with increasing legal conflicts over water,^35^ national assessments documenting the spread of water conflicts across Germany,^27^ and case studies describing recent escalations in conflicts that persisted over long periods e.g., Brauner et al.^16^ Together, these findings indicate that groundwater conflicts are no longer isolated events but are becoming a widespread phenomenon in Germany.

Using text mining techniques, we demonstrated that groundwater-related conflicts can be reliably detected and characterized from news media, with an average accuracy of 0.89 compared to the manual coding of the news. By leveraging increasingly available news archives, we can deliver a spatially explicit analysis of groundwater conflicts without requiring pre-filtered, conflict-specific corpora. Previous studies that leveraged text mining for water-related conflicts relied on conflict-specific documents, such as legal reports or governance documents,^37^^,^^38^^,^^39^ limiting their applicability to contexts where such specialized datasets are available. In contrast, our approach is transferable and scalable across large-scale heterogeneous text resources.^40^^,^^41^ This is particularly important given that most groundwater-conflict research remains based on individual case studies e.g., Brauner et al.,^16^ Koch et al.,^42^ and Steinhäußer^43^ and our approach enables systematic and comparative analyses.

From a technical perspective, our approach is transferable and particularly suited to capturing non-violent conflicts. It can also be used to monitor latent or fuzzy phenomena that are difficult to detect with conventional indicators. This is particularly relevant for non-violent water conflicts, which rarely leave formal administrative or legal traces, making them largely invisible in traditional datasets.^44^ News-based text mining, by contrast, captures public disputes, contestations, and emerging tensions long before they escalate or appear in institutional records, thereby enabling systematic and comprehensive observation of precisely those non-violent conflicts that are otherwise underreported. However, it should be emphasized that applying this approach to other domains requires a sufficiently large news archive with spatiotemporal coverage of the study region, as well as pre-trained word embeddings and sentence transformers to identify conflicts and their drivers.

Our analysis revealed that shifts in the factors associated with conflicts accompany the increasing overall relevance of conflicts. Over time, pollution and environmental protection have declined as dominant themes in conflict reporting, while concerns about agriculture, groundwater scarcity, and drought have intensified significantly. These findings are shared in the literature e.g., Brauner et al.,^16^ Cullmann et al.,^17^ and Kosow et al.^19^ For instance,^19^ describes for Germany “a shift from water resource conflicts linked to industrial pollution to tensions related to climate change effects as drought events”. The diminishing relevance of pollution and water-quality-related conflicts is striking, given that pollutants regularly exceed regulatory thresholds in many groundwater bodies. Yet, this emphasizes that water quality characteristics of groundwater bodies do not translate directly into publicly debated conflict hotspots.^17^^,^^45^ While groundwater quality and quantity are interlinked, water quality-related conflicts, particularly those associated with diffuse pollution such as nitrate, are less likely to escalate into visible conflict hotspots because their impacts can often be mitigated or circumvented and do not immediately affect water availability or consumer prices. In contrast, groundwater scarcity is perceived as more immediate and threatening, which may explain its stronger association with conflict escalation.^46^

These heterogeneous trends were reflected in the four regional clusters across Germany with recurring conflicts. For example, the largest hotspot cluster region in Eastern Germany is characterized by drought-prone basins and declining groundwater recharge.^47^ These stressors were additionally compounded by mining (due to the widespread open-pit lignite mining activities in the area), and industrial activities that created severe groundwater deficits and contamination that persist into the present.^42^^,^^48^^,^^49^^,^^50^ Instead, the southwest Germany cluster has been characterized by contamination (high levels of nitrates and other industrial pollutants) in the early phases of the study period, which decreased afterward as overall pollution became less relevant as a driver of conflict. Hence, conflict levels during the 2018–2022 drought were lower than in other clusters.^51^ These regional differences align with a climatic gradient in Germany, where northeastern regions exhibit systematically lower precipitation and greater drought impacts compared to wetter areas in the southwest.^52^ Yet, the different patterns in hotspot distributions of driver shares highlight the diversity of groundwater-related conflicts across Germany, which are not static but change over time.

Our analyses show that drought periods coincide with increases in reported groundwater conflicts. We found sudden increases in the number of districts affected by conflicts that were temporally aligned with droughts in 2011, 2015, and 2018.^25^ This finding is consistent with previous studies that link droughts to increased conflict levels.^9^^,^^53^ However, these patterns may also reflect other hydroclimatic dynamics. For instance, periods of elevated groundwater levels, particularly in northeastern Germany following 2010, could likewise influence conflict dynamics and spatial patterns independently of drought conditions.^54^ We also found that during the multi-year drought from 2018 to 2022, several regions in northeastern Germany emerged as new hotspots of conflict. In some cases, drought conditions intensified pre-existing tensions. For example, the decades-old groundwater dispute in Hamburg (between the city’s water utility and local residents over pumping) escalated sharply during extreme dryness.^16^ Such patterns align with previous research showing that climate change creates new water scarcity hotspots.^6^^,^^33^^,^^55^ Yet our study offers a unique perspective by highlighting these linkages in non-violent conflicts in historically less conflict-prone areas, rather than relying on single conflict case studies. Spotting new hotspots and the underlying mechanisms contributing to them, both on a larger scale beyond single case studies and in hydro-geological settings, is important because it allows us to evaluate how existing theories of water conflict translate to these novel settings and where they fall short. The linkages between conflicts and droughts that we discern are, however, descriptive rather than causal. Future research, therefore, needs to understand why and how the new hotspots that we uncover occur and to what extent their occurrence is linked to the actual drought hazard, distinguishing drought-driven conflict dynamics from other structural pressures such as institutional path dependencies, local governance capacity, or underlying socio-economic change.^56^^,^^57^^,^^58^

Linking reported conflicts to groundwater quality and quantity variables showed only moderate to low levels of correlation, highlighting that there are multiple interacting pathways and contextual conditions underlying conflicts.^9^^,^^17^ The strongest associations link conflicts to groundwater withdrawal, specifically withdrawal from non-public entities. This suggests that questions of water access, allocation, and control may be more salient conflict factors than groundwater abstraction or recharge alone. In contrast, weak correlations with nitrate pollution and groundwater recharge likely reflect the highly localized, heterogeneous social or hydrological conditions.^7^^,^^9^^,^^10^^,^^54^^,^^59^ Consistent with this interpretation, we observe districts with similar nitrate levels but differing levels of conflict. Based on previous research, pollution may lack the social visibility necessary to trigger political mobilization,^60^ while declining groundwater levels and use restrictions produce highly visible disruptions through their tangible, direct impacts on farmers and citizens. This finding challenges simplistic assumptions that environmental stress directly translates into social conflict, instead emphasizing the context-specific interplay of governance structures, stakeholder relationships, and institutional capacity.^61^^,^^62^^,^^63^ Similarly, the peculiar local combination of hydrogeological and meteorological conditions,^54^ paired with specific water demands and local use patterns, may ultimately determine whether a drought will lead to a significant groundwater conflict. Even with modest correlations, our systematic mapping of conflicts across space and time remains valuable precisely because it reveals where emerging social tensions appear independently of biophysical thresholds.^64^^,^^65^ Such mapping helps identify conflict-prone governance settings that may otherwise remain hidden in traditional hydrogeological datasets.^38^

### Limitations of this study

While the use of news articles can support the large-scale assessment of reported groundwater conflicts, it also introduces biases. Reporting patterns can be influenced by regional differences in media density, editorial priorities, or ownership structures, which may influence the extent to which conflicts are reported across districts. Media coverage may also favor acute drought events over chronic water quality issues like nitrate pollution, which persist without generating current headlines. More broadly, news reporting is affected by reporting biases,^66^ including the potential omission or overemphasis of impacts.^67^ These biases may influence reporting on conflicts across districts and over time. Our analysis relies on the relative groundwater conflict (RGC) metric, which we compute based on the intensity of reported conflicts in media coverage. Importantly, this measure does not count articles; instead, it combines the frequency of articles mentioning conflicts and the prominence of conflict as a topic within them. It reflects the intensity with which groundwater conflicts are reported in the media. Consequently, variation in RGC partly reflects variation in media attention, and the detected hotspots indicate significantly higher levels of reported conflicts, which may not directly reflect the “objective” intensity of conflicts on the ground.

Yet, despite these representational biases, media-derived metrics can still reflect socio-environmental phenomena. Research by Sodoge et al.^68^ on a similar text corpus showed that the impact of drought, as reported in news articles, correlates with external indicators such as crop yields, forest fire reports, or precipitation deficits. Similar to previous research, we attempted to minimize reporting bias by considering a wide range of news outlets (*n* = 260) and removing duplicate news articles stemming from press agencies.^68^ Finally, the growing volume of news articles in our database limits comparisons of conflict intensity over time. The news database contains 11.8 million articles for 2022, while only 3.7 million news articles are available for 2000. Therefore, we focus on comparing geographical hotspots and the relative shares of drivers rather than on absolute numbers.

Further limitations concern the groundwater data sources used to compare our results. The selected datasets on groundwater recharge, pollution, and withdrawal suffer from estimation uncertainties and temporal resolution limitations and generally exhibit high spatial variability. For instance, groundwater withdrawal data are available only at 3-year intervals, while the pollution data represent only the current pollution status, and recharge is taken as a long-term average. Moreover, the small-scale spatial heterogeneity is masked by district-level aggregation. Groundwater recharge results from complex processes that transmit precipitation via infiltration and deeper percolation into aquifers, often with significant time delays and non-linearities. Therefore, groundwater recharge rates are difficult to quantify, usually evaluated as longer-term (monthly or yearly) averages and exhibit significant spatial variability.^54^ This obscures the analysis of localized conflict hotspots by smoothing over critical within-district variation. Considering these limitations, our analysis should be interpreted as identifying broad-scale patterns rather than establishing precise causal relationships. The observed correlations likely underestimate the true relationships due to spatial aggregation effects and temporal mismatches. Beyond these data quality limitations, the selection of indicators was constrained by data availability. While the chosen indicators are well established in the literature on groundwater conflicts in Germany,^17^^,^^69^ they do not capture the full range of pressures potentially relevant to local disputes, such as stored groundwater volumes or contaminants other than nitrate. For future research aimed at better understanding which specific risk factors trigger conflicts, improved data on actual abstractions with higher spatial and temporal resolution, and better estimates of stored groundwater volumes in aquifers will be essential.

## Resource availability

### Lead contact

Requests for further information and resources should be directed and will be fulfilled by the lead contact, Jan Sodoge (jan.sodoge@ufz.de).

### Materials availability

This study did not generate new materials.

### Data and code availability

• Code used in the analysis will be made publicly available immediately after publication.
• All data reported in this publication that is not restricted by copy-right and licenses will be shared by the lead contact upon request.

## Acknowledgments

We thank Jasmin Heilemann for sharing data on groundwater recharge in Germany and Elisie Kåresdotter for sharing literature and data on global water-related conflicts. We also thank Nele Eichler and Maike Reichel for supporting the data annotation of news articles. This project was partially funded through the “Quantification of the effects of adaptation measures on spatiotemporal changes in flood and drought risk (EXTREME-ADAPT)”, an InnoPool project funded by the 10.13039/501100009318Helmholtz Association.

## Author contributions

Conceptualization, J.S., C.K., G.D.B., and M.M.d.B.; methodology, J.S.; software, J.S.; formal analysis, J.S. and J.H.F.; investigation, J.S. and M.M.d.B.; data curation, J.S. and P.E.; writing – original draft, J.S. and M.M.d.B.; writing – review and editing, J.S., C.K., G.D.B., J.H.F., P.E., and M.M.d.B.; visualization, J.S. and M.M.d.B.; supervision, C.K. and M.M.d.B.; funding acquisition, C.K. and M.M.d.B.

## Declaration of interests

The authors declare that they have no known competing financial interests or personal relationships that could have influenced the work reported in this paper. The second affiliation of Jan Sodoge (Technopolis Group) was added per journal guidelines (declaration of interests). The research was, however, conducted exclusively as a researcher with the first affiliation (Helmholtz-Centre for Environmental Research).

## STAR★Methods

### Key resources table

**Table:** 

REAGENT or RESOURCE	SOURCE	IDENTIFIER
**Deposited data**		

GloVe word embeddings	Pennington et al.^77^	https://nlp.stanford.edu/projects/glove/
Groundwater withdrawal data	Statistisches Bundesamt^70^	https://www.umweltbundesamt.de/publikationen/ auswirkung-des-klimawandels-auf-die
Groundwater recharge data	Bundesanstalt für Geowissenschaften und Rohstoffe^71^	https://www.bgr.bund.de/DE/Themen/Wasser/Projekte/abgeschlossen/Beratung/Had/Was_had_abb_gwn1000.html
Nitrate concentration data	Umweltbundesamt^72^	https://www.umweltbundesamt.de/umweltatlas-karte/nitrat-im-grundwasser

**Software and algorithms**		

Concept Mover’s Distance (CMD)	Stoltz et al.^76^	https://gitlab.com/culturalcartography/text2map
BERTopic (zero-shot)	Grootendorst et al.^83^	https://arxiv.org/abs/2203.05794
Getis-Ord Gi∗ (sfdep)	Parry et al.^85^	https://doi.org/10.32614/CRAN.package.sfdep
Structural change detection (strucchange)	Zeileis et al.^87^	https://doi.org/10.32614/CRAN.package.strucchange
LEXpander	Di Natale et al.^74^	https://annadinatale.shinyapps.io/lexpander_app/

### Experimental model and study participant details

No human participants, animals, or cell lines were involved.

### Method details

We combined natural language processing (NLP) with geo-spatial statistics to detect and characterize groundwater conflicts at fine temporal and spatial scales (see Figure S1). News articles served as our primary data source, providing well-edited, locally detailed accounts that capture a wide spectrum of conflicts, including those that do not escalate to violence or legal disputes.^73^ We analyzed a sample of about 12,000 news articles that mention groundwater and conflict-related keywords. First, we measured whether the articles describe groundwater-related conflicts using concept mover’s distance (CMD). Next, we analyzed the drivers of these conflicts by measuring their presence in the article’s text using a zero-shot BERT topic model. We then applied geospatial statistics to detect regional conflict hotspots. Finally, we correlated the reported groundwater conflicts with external variables reflecting groundwater quantity and quality.

#### Text data collection and pre-processing

We considered a corpus of 170 million news articles published between 2000 and 2022. These articles cover 250 German print and online news outlets, including 144 local, 63 regional, and 43 national sources. To identify potentially relevant articles, we filtered the corpus by selecting articles that contained the German term for groundwater (‘*Grundwasser*’) and at least one synonym for the word conflict (*‘Streit’, ‘Konflikt’, ‘Uneinigkeit’, ‘Differenz’, ‘Auseinandersetzung’, ‘Zwist’, ‘Streitfall’, ‘Zank’, ‘Auseinandersetzung’*). This list of synonyms was compiled using synonym dictionaries and co-occurrence analysis.^74^ Based on this search, we retrieved 12,782 potentially relevant articles. We then removed duplicate articles by computing the Jaccard similarity,^75^ considering a >90% similarity threshold, which was based on the distribution of similarities for threshold selection details see.^68^ As a result, we obtained a dataset of 7,898 unique articles that potentially describe groundwater conflicts.

#### Detecting groundwater conflicts from news articles

In this study, we define a “groundwater conflict” as a publicly visible and sustained dispute or controversy among multiple stakeholders over the use, allocation, quality, or protection of groundwater. This definition explicitly excludes private or interpersonal disagreements. Following,^36^ visibility is a core component: conflicts must be publicly visible, manifested through local activism, citizens’ groups, legal actions, or intensive media attention. In short, a conflict must be visible to the public and hence appear in traditional news media to be considered in our study. Similar definitions are used in other work on environmental conflicts that draws on newspaper articles as a primary data source for mapping and monitoring disputes.^36^^,^^44^ This distinguishes our focus from widely used databases such as the Water Conflict Chronology^14^ and the Transboundary Freshwater Disputes Database,^15^ which include only events in which water is a trigger, is used as a weapon, or is a target or casualty of violence.

To assess whether an article reports on groundwater-related conflicts, we used the concept movers distance (CMD) metric.^76^ Unlike traditional binary classification approaches, CMD provides a continuous measure of the prevalence of a theme or concept in a text, offering greater flexibility for capturing ambiguous or latent concepts. In short, CMD measures the extent to which a text engages with a predefined concept by comparing the meanings of its words to the concept under study using pre-trained word embeddings. Embeddings represent words as high-dimensional vectors, where semantic similarity is reflected in spatial proximity.^77^ For instance, the vectors of the terms *mallard* and *duck* are closer to each other than to the word *coffee*. The concept of interest is defined by a seed phrase (or word) that captures its meaning. The CMD score is then calculated by measuring the distance between the seed phrase embedding and the embeddings of words in the target text. Lower distances correspond to higher CMD values, indicating greater semantic similarity between the text and the concept. In this study, we applied the GloVe word embeddings,^77^ a standard model widely applied in CMD-based studies.^76^

We used the term *groundwater conflict* (in German: *Grundwasser Konflikt*) as the seed phrase for the CMD and calculated its similarity to each of the 7,898 unique news articles in our sample. This resulted in a CMD score for each news article, with higher values indicating a closer semantic relationship to the term “groundwater conflict”. The CMD scores ranged from 3.95 to 23.96, with Q1 = −0.67 and Q3 = 0.58 (mean = 0).

We considered articles with a CMD >0 (*n* = 4,(823) to be conflict-related. This threshold was selected based on the manual inspection of articles sampled from deciles of the CMD distribution to identify the point at which articles consistently described groundwater conflicts. To this end, we manually classified each article into 4 categories: no conflict, rather no conflict, rather a conflict, and clear conflict to account for the ambiguities and fuzziness in determining what constitutes a groundwater-related conflict. Using this reference set, we computed accuracy, precision, and recall metrics^78^ to evaluate the CMD performance. We also computed inter-annotator agreement (Krippendorff’s Alpha) to assess the consistency and reliability of the manual classifications.^79^ It should be emphasized that the threshold was only used to exclude clearly irrelevant articles. As such, it is not critical to the analysis itself, since our subsequent analyses sum CMD scores rather than count articles, thereby preserving variation in conflict intensity.

#### Locating groundwater conflicts

For each conflict-related article (i.e., with a CMD >0), we estimated the location of the reported conflict at the district level, using the Nomenclature of Territorial Units for Statistics (NUTS-3 units). This was done following the approach proposed by^68^ and further evaluated by.^80^ First, location entities (i.e., names) were extracted using spaCy’s^81^ named entity recognition and matched with GeoNames to obtain geographic coordinates,^82^ removing ambiguous and overly broad entries (e.g., “the Alps”). Next, we applied hierarchical clustering with a distance-based cutoff to distinguish true impact locations from unrelated mentions (e.g., expert affiliations). Finally, identified locations were mapped to NUTS statistical regions so that each conflict-related article maps to the relevant districts (NUTS-3 units). Conflict-related articles without a German location detected were removed, as they do not cover a specific conflict event.

#### Characterizing the reported drivers of groundwater conflicts

After identifying conflict-related articles mentioning a specific location, we characterized them based on the potential conflict drivers reported in the news. It should be noted that we do not imply a causal relationship between these drivers and the occurrence of conflicts. Instead, they should be understood as factors reported in the media as associated with groundwater-related conflicts. They reflect how conflicts are contextualized in the public discourse. Therefore, the term “driver” should be understood as factors frequently reported in connection with conflicts, not necessarily as proven causal mechanisms.

We derived a typology of such drivers for our case study in Germany by combining a BERT topic model analysis^83^ and existing literature on groundwater conflicts in Germany e.g.,.^17^^,^^27^ The topic model analysis provided an inductive, exploratory approach toward mapping topics in the identified conflict-related articles. Based on both approaches, we compiled a list of drivers, which was refined through informal discussions with co-authors and experts in the field. As a result, we defined seven recurring conflict drivers in Germany (see Table 2).

To detect the presence of these seven drivers in each article, we employed a zero-shot BERT topic model,^83^ which allows for the predefined specification of topics. For each driver, we formulated brief text descriptions (see Table 2). Given BERT’s limitations in handling long texts, articles were segmented into multiple chunks, each approximately 350–400 words long. The chunk size of approximately 350–400 words was determined by the maximum input length of the applied sentence-transformer, thereby ensuring that each text segment provides sufficient context for accurate driver classification. The zero-shot topic model then compared each chunk to the text descriptions of each driver using their semantic (cosine) similarity. Each chunk could be matched to multiple drivers, and a driver was assigned to a given text chunk if the cosine similarity exceeded 0.65; consequently, multiple drivers could be assigned to the same chunk when this threshold was exceeded for more than one driver. This threshold was defined based on general recommendations for this method and qualitative inspection of the data.^83^ After classifying each text chunk, we calculated the relative share of each driver as a proportion of the total number of drivers detected in each article. We evaluated our approach by manually reviewing 100 randomly selected articles for each driver and assessing whether the driver was indeed present in the text chunks. Classification performance was measured using accuracy, precision, and recall.^78^ We also tested different text descriptions. The resulting similarity scores remained highly correlated, suggesting that model performance was stable across different phrasings of the driver definition.

#### Geo-statistical analyses of conflict hotspots and their drivers

To identify regional and temporal hotspots of reported groundwater conflicts, we aggregated the conflict-related articles (i.e., those with a CMD >0) by district and year. We introduce a metric called Reported Groundwater Conflicts (RGC), which sums the CMD scores of conflict-related articles for each district and year. RGC thus captures both the frequency and the prominence of conflict reporting. Articles in which conflicts are more strongly represented contribute more to the total. As a result, the same RGC score can result from fewer articles with high CMD scores or more articles with low CMD scores.

After calculating the RGC, we used a Getis-Ord Gi∗ analysis to detect regions with significantly high (referred to as hotspots) or low (referred to as coldspots) RGC scores by comparing observed spatial patterns to a random distribution.^84^^,^^85^ We applied this method separately for each year to determine whether conflict hotspots are recurring, declining, or emerging.^86^ This approach allowed us to identify regional clusters that consistently showed significantly higher RGC volumes. As a robustness check, we tested alternative aggregations of the conflict measure, including summing CMD scores above higher thresholds (0.1, 0.3, 0.(5) and counting articles above zero, finding hotspot agreement rates of 97.7–98.7% and 92–98.3% respectively, confirming that the main spatial patterns are stable across aggregation choices.

To identify broader regional clusters beyond individual hotspots, we mapped the frequency with which each district was classified as a hotspot over the 23-year study period (2000–2022). We then grouped neighboring districts with consistently high frequencies into larger regions referred to as ‘hotspot clusters’ in the subsequent analysis. This step was based on visual inspection of contiguous high-frequency areas from the Getis-Ord Gi∗ analysis and expert judgment combining socio-economic and hydrological perspectives. This allowed us to identify and analyze recurring conflict patterns at a regional scale. We acknowledge that this delineation step involves an element of subjectivity, as the statistical methods alone do not determine how annual hotspot results should be grouped into broader regional clusters.

To better understand the temporal and spatial distribution of conflicts and their drivers across regional clusters, we applied two analyses. For temporal dynamics, we used a breakpoint detection algorithm^87^ to identify potential sudden shifts in the temporal occurrence of reported conflicts. This algorithm statistically tests for structural breaks in time series data, allowing us to detect years when conflict prevalence changed significantly rather than evolving gradually. For spatial differences, we used Moran’s I, a measure that tests whether conflicts or their drivers cluster in space rather than being randomly distributed. For both temporal and spatial comparisons, we compared the prevalence of different conflict drivers between regions and over time using Mann-Whitney statistical tests. These enable pairwise comparisons between districts or years and do not require specific data distributions.^88^ For its implementation, we used the drivers’ relative shares for each conflict-related article, grouped by district or year.

#### Evaluating the effect of the 2018–2022 drought on groundwater conflicts

To investigate changes in conflict patterns from before (2000–2017) to during the multi-year drought period (2018–2022),^89^ we conducted spatial and driver-focused analyses. First, we calculated the change in conflict intensity (measured via RGC) between the two periods across districts. Specifically, we calculated the relative conflict prevalence for each district for both the pre-drought period and the multi-year drought. We then standardized these values to make them comparable across periods, allowing us to assess which districts experienced a higher conflict intensity during the drought. This approach focuses on relative changes rather than absolute counts, thereby avoiding media bias caused by the overall increase in reported conflicts. As such, it allows highlighting areas where drought conditions coincided with disproportionately higher conflict intensity. Second, we analyzed changes in the prevalence of conflict drivers between the two periods using Mann-Whitney tests. For each of the seven drivers, we tested whether its relative share differed significantly between the two periods. Analyses were conducted both for Germany on an aggregated level and at the district level.

#### Correlating reported groundwater conflicts and groundwater withdrawal, recharge, and pollution

We evaluated the link between reported groundwater conflicts and physical groundwater variables often associated with them: groundwater withdrawal, recharge, and pollution (see Table 1). Groundwater withdrawal was selected as it directly reflects the extraction pressure on an aquifer, which has been studied as a driver of conflicts.^21^^,^^90^ We considered both public and non-public groundwater withdrawal. Public water withdrawal denotes abstraction for the public water supply by municipalities or utilities serving the general population, while non-public water withdrawal refers to commercial and industrial uses such as mining and manufacturing. Groundwater recharge rates were chosen because they indicate baseline hydrogeological capacity and natural replenishment potential of aquifers. Regions with inherently lower recharge capacity may be more vulnerable to scarcity-related conflicts, particularly when temporal stressors such as droughts reduce actual recharge below these baseline levels.^5^^,^^7^ Nitrate concentration was selected as an indicator of groundwater quality degradation primarily from agricultural sources, a widespread problem that has been documented as a significant contributor to groundwater-related disputes in Germany.^17^^,^^69^^,^^91^ Nitrate concentrations in groundwater reflect the cumulative effects of decades of excess reactive nitrogen inputs and therefore represent a long-term pressure that responds much more slowly to short-term meteorological variability such as droughts.^92^ Yet, we acknowledge that nitrate is not a proxy for groundwater quality degradation more broadly, as other pollutants, such as pesticides, may also contribute to local disputes.

To ensure comparability across datasets with varying spatial resolutions, we aggregated all indicators to the NUTS-2 level by grouping data by district and analysis period (2000–2022 and 2018–2022) and calculating mean values for each district-period combination. We then correlated these aggregated indicators with the sum of reported conflict scores for each district and period to link with the RGC. This aggregation approach allows us to examine whether regions with higher groundwater stress or quality issues also experience more intense reported conflicts. Given the nonnormal distribution of the data, we employed Spearman’s correlation coefficient to test associations between groundwater indicators and the RGC. Specifically, we computed correlations between the number of reported conflicts per region and each groundwater indicator for both the entire study period (2000–2022) and the multi-year drought period (2018–2022).

### Quantification and statistical analysis

Statistical analyses were performed in R. To detect groundwater conflicts from news articles, we used the CMD metric^76^ with GloVe word embeddings.^77^ Classification performance was evaluated using accuracy, precision, and recall,^78^ and inter-annotator agreement was assessed using Krippendorff’s Alpha.^79^ Conflict drivers were classified using a zero-shot BERT topic model^83^ with a cosine similarity threshold of 0.65. To identify regional conflict hotspots, we computed the RGC metric (summing CMD scores per district and year) and applied Getis-Ord Gi∗ analysis^84^^,^^85^ separately for each year. Temporal structural breaks were identified using the strucchange package.^87^ Spatial clustering of drivers was assessed using Moran’s I. Pairwise comparisons of driver prevalence across regions and periods used Mann-Whitney tests.^88^ Associations between RGC and physical groundwater indicators were assessed using Spearman’s rank correlation coefficient, computed separately for the full study period (2000–2022) and the drought period (2018–2022).

## References

[1] Gorelick S.M., Zheng C. (2015). Global change and the groundwater management challenge. Water Resour. Res..

[2] Kåresdotter E., Skoog G., Pan H., Kalantari Z. (2023). Water-related conflict and cooperation events worldwide: A new dataset on historical and change trends with potential drivers. Sci. Total Environ..

[3] Mukherjee A., Scanlon B.R., Aureli A., Langan S., Guo H., McKenzie A.A. (2020).

[4] Institute P. (2024). Water Conflict Chronology. Pacific Institute.

[5] Robins N.S., Fergusson J. (2014). Groundwater scarcity and conflict – managing hotspots. Earth Perspect..

[6] Huggins X., Gleeson T., Villholth K.G., Rocha J.C., Famiglietti J.S. (2024). Groundwaterscapes: A global classification and mapping of groundwater’s large-scale socioeconomic, ecological, and Earth system functions. Water Resour. Res..

[7] Jarvis W.T. (2014).

[8] Jarvis W.T., Villholth K.G., Lopez-Gunn E., Conti K., Garrido A., Van Der Gun J. (2017). Advances in groundwater governance.

[9] Ide T., Lopez M.R., Fröhlich C., Scheffran J. (2021). Pathways to water conflict during drought in the MENA region. J. Peace Res..

[10] Suleymanov F. (2024). A review of the multifaceted relationship between drought dynamics and conflicts. Hydrol. Process..

[11] Li X., Zhang X., Wang S. (2021). Managing conflicts and equitability in hierarchical decision making for water resources planning under fuzzy uncertainty: A case study of Yellow River, China. J. Hydrol.: Reg. Stud..

[12] Benjaminsen T.A., Maganga F.P., Abdallah J.M. (2009). The Kilosa killings: Political ecology of a farmer–herder conflict in Tanzania. Dev. Change.

[13] Bassett T.J. (1988). The political ecology of peasant-herder conflicts in the northern Ivory Coast. Ann. Assoc. Am. Geogr..

[14] Gleick, P.H. (2000). Water Conflict Chronology. The World’s Water 2008-2009: The Biennial Report on Freshwater Resources pp. 151–196.

[15] Wolf A.T. (1999). The Transboundary Freshwater Dispute Database Project. Water Int..

[16] Brauner S. (2024). “Water conflicts: more than conflicts over distribution? Assessing conflict structures with cases from Germany”. Discov. Water.

[17] Cullmann A., Sundermann G., Wägner N., von Hirschhausen C.R., Kemfert C. (2022). Water resources in Germany: Increasingly polluted and regionally overused. DIW weekly report.

[18] Bluemling, B., and Horstkoetter, M. (2006). Agricultural Groundwater Protection through Groundwater Co-operations in Lower Saxony, Germany,–a Multi Stakeholder Task.

[19] Kosow H., Brauner S., Brumme A., Hauser W., Hölzlberger F., Moschner J., Rübbelke D., Vögele S., Weimer-Jehle W. (2024). Uncharted water conflicts ahead: mapping the scenario space for Germany in the year 2050. Front. Water.

[20] Yoffe S., Fiske G., Giordano M., Giordano M., Larson K., Stahl K., Wolf A.T. (2004). Geography of international water conflict and cooperation: Data sets and applications. Water Resour. Res..

[21] Llamas M.R., Martínez-Santos P. (2005). Intensive groundwater use: silent revolution and potential source of social conflicts. American Society of Civil Engineers.

[22] Hensel P.R., McLaughlin Mitchell S., Sowers T.E. (2006). Conflict management of riparian disputes. Polit. Geogr..

[23] Carvalho T.M.N., Zscheischler J., Kuhlicke C., de Brito M.M. (2024). A global database of natural hazards impacts reported in the scientific literature. Tech. Rep. Copernicus Meetings.

[24] von Uexkull N., Croicu M., Fjelde H., Buhaug H. (2016). Civil conflict sensitivity to growing-season drought. Proc. Natl. Acad. Sci. USA.

[25] Boeing F., Rakovec O., Kumar R., Samaniego L., Schrön M., Hildebrandt A., Rebmann C., Thober S., Müller S., Zacharias S. (2022). High-resolution drought simulations and comparison to soil moisture observations in Germany. Hydrol. Earth Syst. Sci..

[26] Rakovec O., Samaniego L., Hari V., Markonis Y., Moravec V., Thober S., Hanel M., Kumar R. (2022). The 2018–2020 Multi-Year Drought Sets a New Benchmark in Europe. Earths Future.

[27] Stein U., Tröltzsch J. (2024). Auswirkung des Klimawandels auf die Wasserverfügbarkeit - Anpassung an Trockenheit und Dürre in Deutschland. Umweltbundesamt.

[28] Tröltzsch J., Stein U., Vidaurre R., Bueb B., Schritt H., Flörke M., Wriege-Bechtold A., Herrmann F. (2021).

[29] LAWA (2023). Positionspapier zum Umgang mit Zielkonflikten bei der Anpassung der Wasserwirtschaft an den Klimawandel. Bund/Länder-Arbeitsgemeinschaft Wasser (LAWA).

[30] OECD (2018). Environment database: freshwater abstractions. https://stats.oecd.org/Index.aspx?DataSetCode=WATER_ABSTRACT.

[31] Riedel T., Weber T.K.D. (2020). The influence of global change on Europe’s water cycle and groundwater recharge. Hydrogeol. J..

[32] Wunsch A., Liesch T., Broda S. (2022). Deep learning shows declining groundwater levels in Germany until 2100 due to climate change. Nat. Commun..

[33] McNamara I., Flörke M., Uschan T., Baez-Villanueva O.M., Herrmann F. (2024). Estimates of irrigation requirements throughout Germany under varying climatic conditions. Agric. Water Manag..

[34] Heilemann J., Klassert C., Samaniego L., Thober S., Marx A., Boeing F., Klauer B., Gawel E. (2024). Projecting impacts of extreme weather events on crop yields using LASSO regression. Weather Clim. Extrem..

[35] Joeres A., Steeger G., Huth K., Jacobsen M., Donheiser M. (2022). Ausgetrocknet - Deutschland kämpft um Wasser. https://correctiv.org/aktuelles/klimawandel/2022/06/14/klimawandel-konflikt-um-wasser-in-deutschland/?lang=de.

[36] Otto A., Hornberg A., Thieken A. (2018). Local controversies of flood risk reduction measures in Germany. An explorative overview and recent insights. J. Flood Risk Manag..

[37] Herrera M., Candia C., Rivera D., Aitken D., Brieba D., Boettiger C., Donoso G., Godoy-Faúndez A. (2019). Understanding water disputes in Chile with text and data mining tools. Water Int..

[38] Lee J.h., Kim D.k. (2020). Mapping environmental conflicts using spatial text mining. Land.

[39] Emmanuel Cookey P., Darnsawasdi R., Ratanachai C. (2017). Text mining analysis of institutional fit of Lake Basin water governance. Ecol. Indic..

[40] Xu G., Meng Y., Chen Z., Qiu X., Wang C., Yao H. (2019). Research on topic detection and tracking for online news texts. IEEE Access.

[41] Smith D.A. (2002). Proceedings of the 25th annual international ACM SIGIR conference on Research and development in information retrieval.

[42] Koch H., Kaltofen M., Grünewald U., Messner F., Karkuschke M., Zwirner O., Schramm M. (2005). Scenarios of water resources management in the Lower Lusatian mining district, Germany. Ecol. Eng..

[43] Steinhäußer R., Siebert R., Steinführer A., Hellmich M. (2015). National and regional land-use conflicts in Germany from the perspective of stakeholders. Land Use Policy.

[44] Bengston D.N., Fan D.P. (1999). Conflict Over Natural Resource Management: A Social Indicator Based on Analysis of Online News Media Text. Soc. Nat. Resour..

[45] Jutglar K., Hellwig J., Stoelzle M., Lange J. (2021). Post-drought increase in regional-scale groundwater nitrate in southwest Germany. Hydrol. Process..

[46] Karandish F., Liu S., De Graaf I. (2025). Global groundwater sustainability: A critical review of strategies and future pathways. J. Hydrol..

[47] Francke T., Heistermann M. (2025). Groundwater recharge in Brandenburg is declining – but why?. Nat. Hazards Earth Syst. Sci..

[48] Grünewald U. (2001). Water resources management in river catchments influenced by lignite mining. Ecol. Eng..

[49] Gerwin W., Raab T., Birkhofer K., Hinz C., Letmathe P., Leuchner M., Roß-Nickoll M., Rüde T., Trachte K., Wätzold F., Lehmkuhl F. (2023). Perspectives of lignite post-mining landscapes under changing environmental conditions: what can we learn from a comparison between the Rhenish and Lusatian region in Germany?. Environ. Sci. Eur..

[50] Somogyvári M., Brill F., Tsypin M., Rihm L., Krueger T. (2025). Regional-scale groundwater analysis with dimensionality reduction. Nat. Hazards Earth Syst. Sci..

[51] Lütkemeier R., Kuhn D., Söller L. (2025). Grundwasserstress in Deutschland: Überblicksstudie – Struktureller und akuter Grundwasserstress durch öffentliche und nichtöffentliche Entnahmen auf Ebene der Landkreise. Technical Report BUND – Bund für Umwelt und Naturschutz Deutschland e.

[52] Jung C., Schindler D. (2019). Precipitation Atlas for Germany (GePrA). Atmosphere.

[53] Unfried K., Kis-Katos K., Poser T. (2022). Water scarcity and social conflict. J. Environ. Econ. Manag..

[54] Ebeling P., Musolff A., Kumar R., Hartmann A., Fleckenstein J.H. (2025). Groundwater head responses to droughts across Germany. Hydrol. Earth Syst. Sci..

[55] Leijnse M., F P Bierkens M., H M Gommans K., Lin D., Tait A., Wanders N. (2024). Key drivers and pressures of global water scarcity hotspots. Environ. Res. Lett..

[56] Buhaug H., Von Uexkull N. (2021). Vicious Circles: Violence, Vulnerability, and Climate Change. Annu. Rev. Environ. Resour..

[57] Nhim T., Richter A. (2022). Path dependencies and institutional traps in water governance – Evidence from Cambodia. Ecol. Econ..

[58] Raja Ariffin R.N., Sawon S., Abd Rahman N.H., Hanafi H., Zahari R.K. (2024). Contextualizing institutional capacity in water governance framework: a literature review. Water Policy.

[59] Barreteau O., Caballero Y., Hamilton S., Jakeman A.J., Rinaudo J.D. (2016). Disentangling the complexity of groundwater dependent social-ecological systems. Integrated Groundwater Management: Concepts, Approaches and Challenges.

[60] Gould K.A. (1993). Pollution and perception: Social visibility and local environmental mobilization. Qual. Sociol..

[61] Wiering M., Kirschke S., Akif N.U. (2023). Addressing diffuse water pollution from agriculture: Do governance structures matter for the nature of measures taken?. J. Environ. Manag..

[62] Theesfeld I. (2010). Institutional Challenges for National Groundwater Governance: Policies and Issues. Groundwater.

[63] Petersen-Perlman J.D., Megdal S.B., Gerlak A.K., Wireman M., Zuniga-Teran A.A., Varady R.G. (2018). Critical Issues Affecting Groundwater Quality Governance and Management in the United States. Water.

[64] Rodríguez-Labajos B., Martínez-Alier J. (2015). Political ecology of water conflicts. WIREs Water.

[65] Di Baldassarre G., Viglione A., Carr G., Kuil L., Yan K., Brandimarte L., Blöschl G. (2015). Debates - Perspectives on socio-hydrology: Capturing feedbacks between physical and social processes. Water Resour. Res..

[66] Rodrigo-Ginés F.J., Carrillo-de Albornoz J., Plaza L. (2024). A systematic review on media bias detection: What is media bias, how it is expressed, and how to detect it. Expert Syst. Appl..

[67] Engelmann I. (2010). Journalistische Instrumentalisierung von nachrichtenfaktoren. Medien Kommunikationswiss. M K.

[68] Sodoge J., Kuhlicke C., de Brito M.M. (2023). Automatized spatio-temporal detection of drought impacts from newspaper articles using natural language processing and machine learning. Weather Clim. Extrem..

[69] Kirschke S., Häger A., Kirschke D., Völker J. (2019). Agricultural nitrogen pollution of freshwater in Germany. The governance of sustaining a complex problem. Water.

[70] Statistisches Bundesamt, G (2025). Genesis-Online - Wassergewinnung. https://www-genesis.destatis.de/datenbank/online.

[71] Bundesanstalt für Geowissenschaften und Rohstoffe, B. . Mittlere jährliche Grundwasserneubildung von Deutschland. . URL: https://www.bgr.bund.de/DE/Themen/Wasser/Projekte/abgeschlossen/Beratung/Had/Was_had_abb_gwn1000.html..

[72] Umweltbundesamt (2024). Nitrat im Grundwasser. https://www.umweltbundesamt.de/umweltatlas-karte/nitrat-im-grundwasser.

[73] Wei J., Wei Y., Tian F., Nott N., de Wit C., Guo L., Lu Y. (2021). News media coverage of conflict and cooperation dynamics of water events in the Lancang–Mekong River basin. Hydrol. Earth Syst. Sci..

[74] Di Natale A., Garcia D. (2024). LEXpander: Applying colexification networks to automated lexicon expansion. Behav. Res. Methods.

[75] Mullen L. (2016). Textreuse: Detect Text Reuse and Document Similarity. R package version 0.1.4.9000.

[76] Stoltz D.S., Taylor M.A. (2019). Concept mover’s distance: Measuring concept engagement via word embeddings in texts. J. Comput. Soc. Sci..

[77] Pennington J., Socher R., Manning C.D. (2014). Proceedings of the 2014 conference on empirical methods in natural language processing (EMNLP).

[78] Tharwat A. (2021). Classification assessment methods. Appl. Comput. Inform..

[79] Krippendorff K. (2004). Reliability in content analysis: Some common misconceptions and recommendations. Hum. Commun. Res..

[80] Madruga de Brito M., Sodoge J., Kreibich H., Kuhlicke C. (2025). Comprehensive assessment of flood socioeconomic impacts through text-mining. Water Resour. Res..

[81] Honnibal M., Montani I. (2017). spaCy 2: Natural language understanding with Bloom embeddings, convolutional neural networks and incremental parsing. To appear.

[82] GeoNames (2022). GeoNames. https://www.geonames.org/.

[83] Grootendorst M. (2022). BERTopic: Neural topic modeling with a class-based TF-IDF procedure. arXiv.

[84] Getis A., Ord J.K. (1992). The Analysis of Spatial Association by Use of Distance Statistics. Geogr. Anal..

[85] Parry, J., and Locke, D. (2022). sfdep: Spatial Dependence for Simple Features. . URL: https://CRAN.R-project.org/package=sfdep.doi:10.32614/CRAN.package.sfdep..

[86] Harris N.L., Goldman E., Gabris C., Nordling J., Minnemeyer S., Ansari S., Lippmann M., Bennett L., Raad M., Hansen M., Potapov P. (2017). Using spatial statistics to identify emerging hot spots of forest loss. Environ. Res. Lett..

[87] Zeileis A., Leisch F., Hornik K., Kleiber C. (2001).

[88] McKnight P.E., Najab J. (2010).

[89] Sodoge J., Kuhlicke C., Mahecha M.D., de Brito M.M. (2023). Text-mining uncovers the unique dynamics of socio-economic impacts during multi-year drought. Nat. Hazards Earth Syst. Sci.

[90] Llamas M.R., Garrido A. (2007). Lessons from intensive groundwater use in Spain: economic and social benefits and conflicts. The Agricultural Groundwater Revolution: Opportunities and Threats to Development.

[91] Junior R.V., Varandas S., Fernandes L.S., Pacheco F. (2014). Groundwater quality in rural watersheds with environmental land use conflicts. Sci. Total Environ..

[92] Kyte E., Cey E., Hrapovic L., Hao X. (2023). Nitrate in shallow groundwater after more than four decades of manure application. J. Contam. Hydrol..

