# Supplementary material for: Emerging hotspots of groundwater conflicts during droughts in Germany
Source: iScience. 2026 Jun 11;29(7):116338. doi: 10.1016/j.isci.2026.116338 (PMC13276450; doi:10.1016/j.isci.2026.116338)
Supplement: Document S1. Figures S1–S3 [file mmc1.pdf]

iScience, Volume *29*

Supplemental information

Emerging hotspots of groundwater conflicts during droughts in Germany

Jan Sodoge, Christian Kuhlicke, Giuliano Di Baldassarre, Jan H. Fleckenstein, Pia Ebeling, and Mariana Madruga de Brito

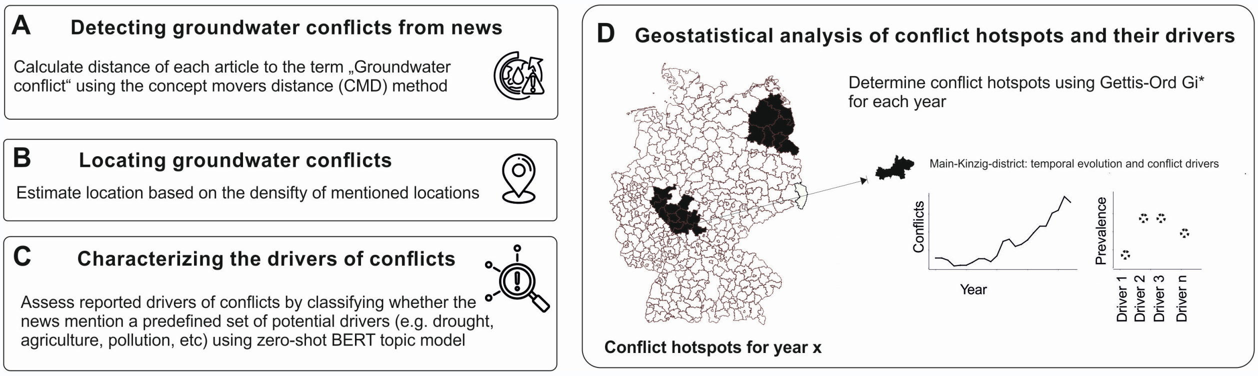

Figure S1: Overview of the text-mining and geo-spatial statistics approach used to detect and characterize groundwater conflicts from a large corpus of news articles.

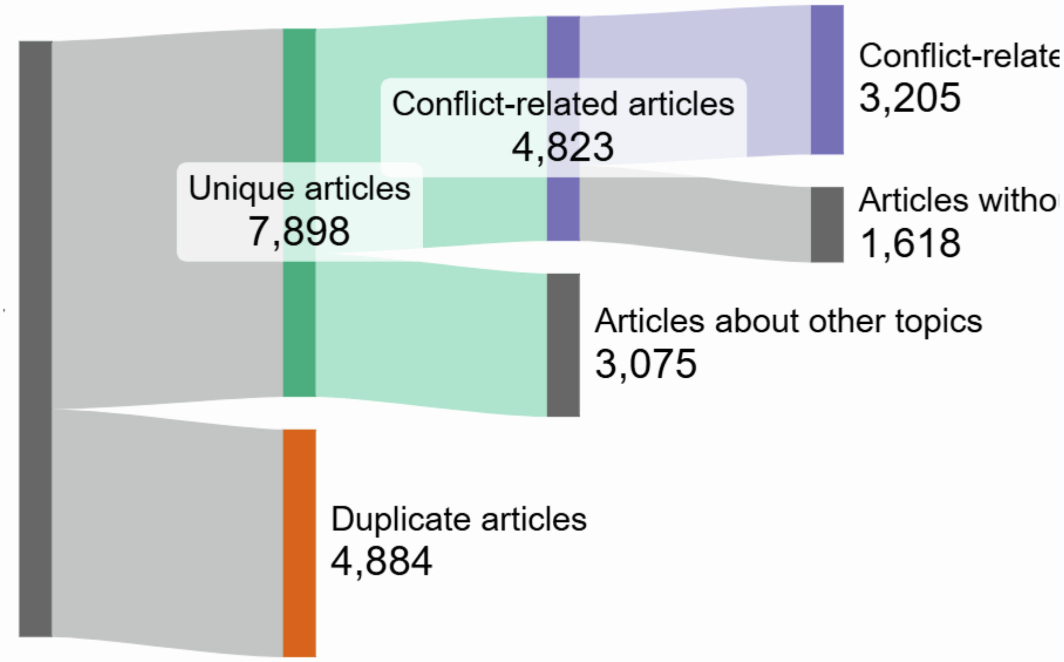

Figure S2: Sankey plot with the steps for the identification of news articles mentioning groundwater conflicts occurring in specific locations.

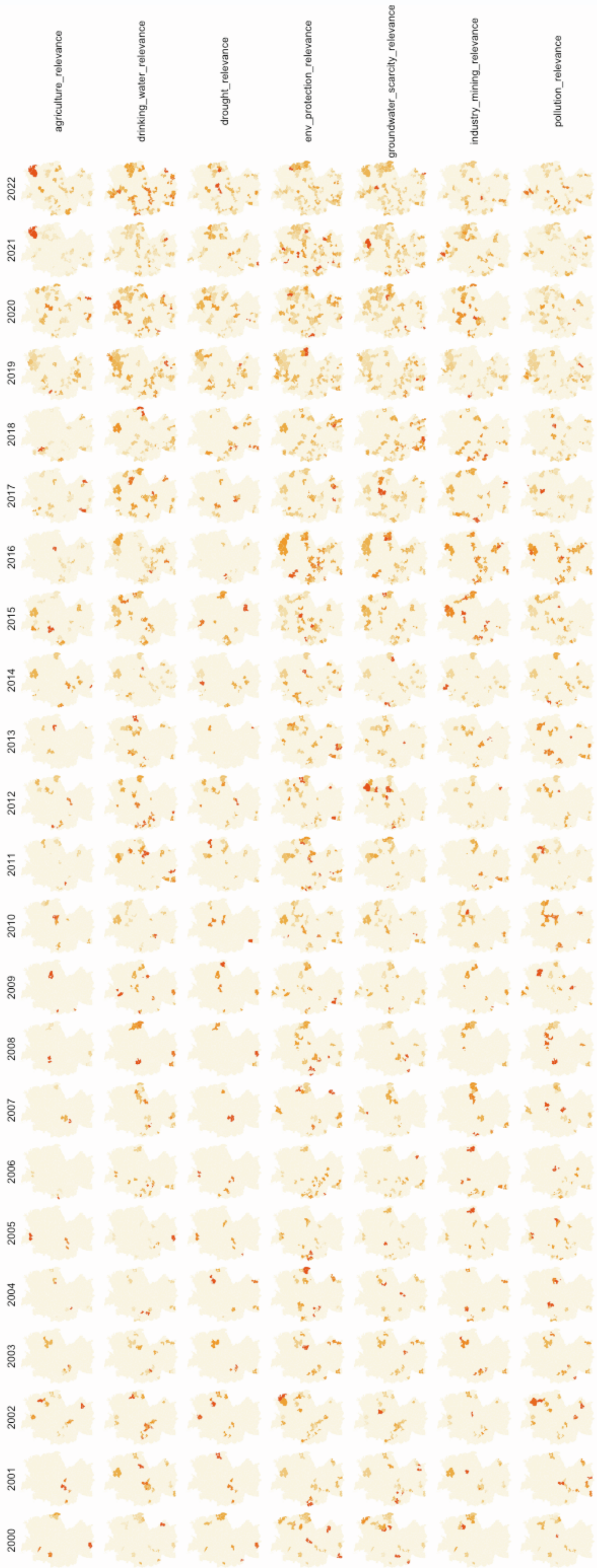

Figure S3: Relative prevalence of reported drivers in groundwater-conflict related articles per each year. Red colors (logarithmic-scaling) indicate stronger prevalence
